# Hyperglycemia is associated with cardiac complications in elderly nondiabetic patients receiving total parenteral nutrition

**DOI:** 10.1097/MD.0000000000009537

**Published:** 2018-02-09

**Authors:** Jinling Ma, Meng Gao, Rong Pan, Lei He, Lei Zhao, Jie Liu, Hongbin Liu

**Affiliations:** a Department of Geriatric Cardiology; b Department of Geriatric Nephrology, Chinese PLA General Hospital, National Clinical Research Center for Geriatric Diseases, Beijing; c Department of Neurology, Dancheng People's Hospital, Henan; d Department of Endocrinology, Chinese PLA 305 Hospital; e Department of Emergency, Chinese PLA General Hospital, National Clinical Research Center for Geriatric Diseases, Beijing, China.

**Keywords:** elderly, mean blood glucose level, total parenteral nutrition

## Abstract

Adverse outcomes have been associated with hyperglycemia in patients receiving total parenteral nutrition (TPN). The relationship may be characteristic in elderly patients. However, limited data are available about the relationship between TPN-related hyperglycemia and cardiac adverse outcome in elderly patients without previously known diabetes. This study aims to identify whether there is an association between hyperglycemia and 45-day cardiac adverse outcomes in critically and noncritically ill elderly nondiabetic patients receiving TPN.

Outcome measures of 45-day cardiac complications after receiving TPN were recorded from a retrospective review of 1517 medical and surgical elderly patients. The mean glucose levels were significantly higher in patients with cardiac complications than in patients without cardiac complications (*P* < .001). In multivariate logistic regression analysis adjusting for age, gender, comorbidities, and medications, higher mean blood glucose levels were independently associated with increased 45-day cardiac complications (odds ratio, 1.62; 95% confidence interval, 1.453–1.816; *P* < .001). Furthermore, Kaplan–Meier event-free survival curves demonstrated that patients with mean blood glucose level ≥11.1 mmol/L had worse cardiac complications event-free survival compared with those mean blood glucose level <11.1 mmol/L during 45 days after receiving TPN.

This study showed that TPN-induced hyperglycemia was associated with increased risk of cardiac complications in both critically and noncritically ill elderly patients without a history of diabetes.

## 1 Introduction

Parenteral nutrition has been well established to have a beneficial effect in improving the nutritional status of hospitalized malnourished patients.^[1]^ Hyperglycemia is a frequent complication during total parenteral nutrition (TPN) therapy,^[2–8]^ independent of a history of diabetes.^[1,9]^ The risk factors for developing hyperglycemia responses to TPN in the absence of diabetes included age and other factors.^[7,9]^Adverse outcomes and increased mortality have been associated with hyperglycemia in patients receiving TPN.^[9–11]^ Moreover, some studies revealed that hyperglycemia in TPN patients is a risk factor for the development of cardiac complications.^[3–5,7,11]^ The relationship may be characteristic in elderly patients with multiple comorbidities, baseline functional status impairment, and a heterogeneous diagnosis. However, limited data are available about the relationship between TPN-related hyperglycemia and cardiac adverse outcome in elderly patients without previously known diabetes.

Aging is characterized by chronic low-grade inflammation that is involved in the pathophysiology and development of many age-related disorders. Aging patients in hospitals have increased risk of cardiac events because of multiple comorbidities. This study aims to identify whether there is an association between hyperglycemia and 45-day cardiac complications in elderly critically and noncritically ill nondiabetic patients receiving TPN.

## 2 Methods

### 2.1 Study population

The current study was a retrospective review of 1517 medical and surgical patients without previously known diabetes aged 60 years and over receiving TPN as a sole source of nutrition between December 2007 and December 2014 at the Chinese PLA General Hospital and Dancheng People's Hospital. Patients who received TPN for a minimum of 7 days were included in the study. The exclusion criteria included lack of detailed blood glucose measurement records during TPN treatment, a baseline blood glucose level ≥11.1 mmol/L before starting TPN, receiving TPN therapy for fewer than 7 days, and currently receiving corticosteroid therapy. In addition, patients were excluded if they were receiving parenteral nutrition together with enteral nutrition. The study was approved by the medical ethics committee of the 2 hospitals.

We collected information on the daily blood glucose levels measured by either capillary or plasma glucose level and calculated the mean blood glucose levels during the duration of the TPN therapy from the hospitals’ database. The medical histories, characteristics, current medications, and demographic data of the patients were also obtained. Mean blood glucose values were calculated for each patient with the total number of readings. The TPN formula at the 2 hospitals was provided as a total nutrient admixture solution containing carbohydrates, proteins, and lipids. Outcome measures of the cardiac complications during 45 days after receiving TPN were recorded from the medical record and by contacting each patient or family individually by telephone. Cardiac complications were defined as an acute coronary syndrome or acute congestive heart failure. Acute coronary syndrome was defined according to the new universal definition.^[12]^ The diagnosis of acute congestive heart failure was based on the guidelines of the European Society of Cardiology. No patient was lost during the follow-up period. Hyperglycemia was defined as mean blood glucose level ≥11.1 mmol/L. These groups were compared in terms of the cardiac complications during the 45 days after receiving TPN.

### 2.2 Statistical analysis

The categorical variables were presented as proportions (percentages). Normally distributed continuous data were expressed as mean ± SD. Categorical variables were compared using χ^2^ tests. Logistic regression analysis was used to predict the prevalence of 45-day cardiac complications, with adjustments for age, sex, comorbidities, and medication. The results were presented as adjusted odds ratios and their 95% confidence interval. A Kaplan–Meier analysis was performed to assess event-free survival. All data were processed using the PASW (version 18.0; SPSS, Chicago, IL). A *P* value <0.05 was considered statistically significant.

## 3 Results

A total of 1517 medical and surgical patients 60 years of age or older without a previous diagnosis of diabetes receiving TPN as a sole source of nutrition were included in the study. During the 45-day TPN therapy follow-up period, the overall cardiac complications were 8.2% (125 of 1517 patients). According to the mean blood glucose levels during TPN, the population was divided into 2 groups: mean blood glucose level ≥11.1 mmol/L (n = 127, 8.4%) and mean blood glucose level <11.1 mmol/L (n = 1390, 91.6%). The cardiac complications during the 45-day TPN therapy was 37.8% in the patients with mean blood glucose level ≥11.1 mmol/L and 5.5% in the patients with mean blood glucose level <11.1 mmol/L. The results showed that the 45-day cardiac complications were significantly higher in the patients with mean blood glucose level ≥11.1 mmol/L (37.8% vs 5.5%, *P* < .001).

**Figure 1:**
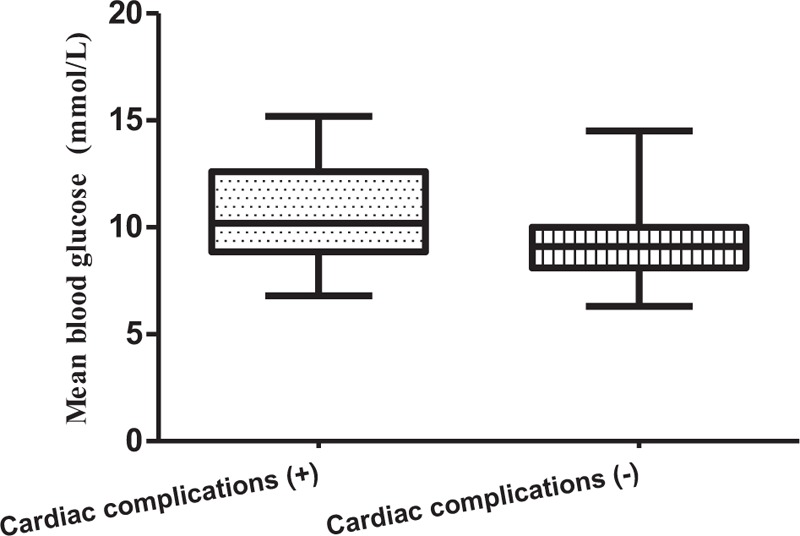
Differences in mean blood glucose levels between the patients.

In the further investigation of the differences between cardiac complications and noncardiac complications during the TPN treatment, we found that the mean glucose levels were significantly higher in the patients with cardiac complications than in those without cardiac complications (*P* < .001) (Table 1). Moreover, differences were found in age and history of coronary heart disease between the 2 groups. No significant differences were found in sex, history of hypertension, atrial fibrillation, chronic obstructive pulmonary disease, malignancy, chronic renal insufficiency, and medications. Mean blood glucose levels in patients with cardiac complications were higher than in patients without cardiac complications (*P* < .001, Fig. 1.).

**Table 1:**
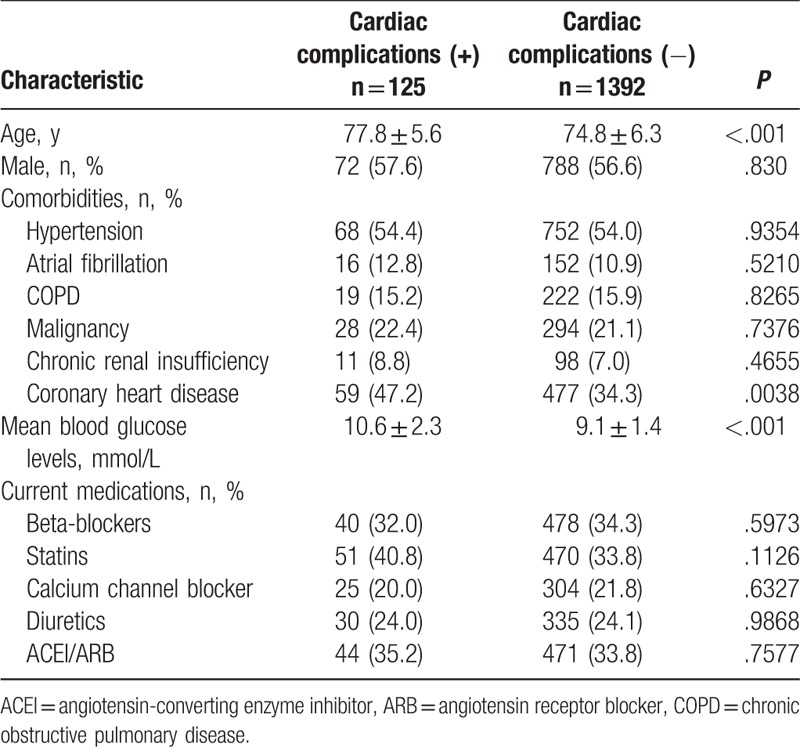
Clinical characteristics according to the occurrence of 45-day cardiac complications.

In the analysis of multivariate logistic regression adjusting for age, gender, comorbidities, and medications, higher mean blood glucose levels were independently associated with increased 45-day cardiac complications (odds ratio 1.62, 95% confidence interval 1.453–1.816, *P* < .001) (Table 2). The result showed that higher mean blood glucose remained an independent predictor for the development of 45-day cardiac complications.

**Table 2:**
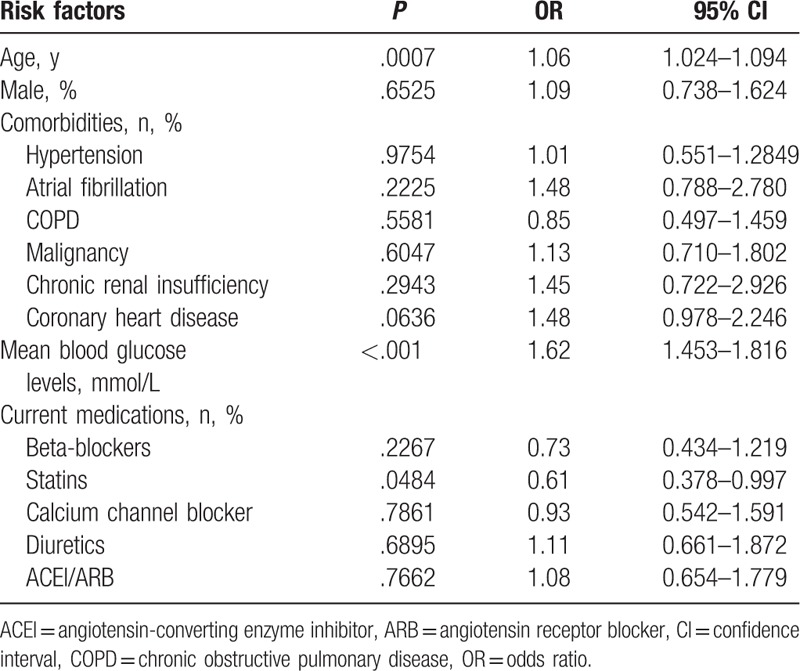
Risk factors associated with 45-day cardiac complications in a multivariate logistic regression analysis.

**Figure 2:**
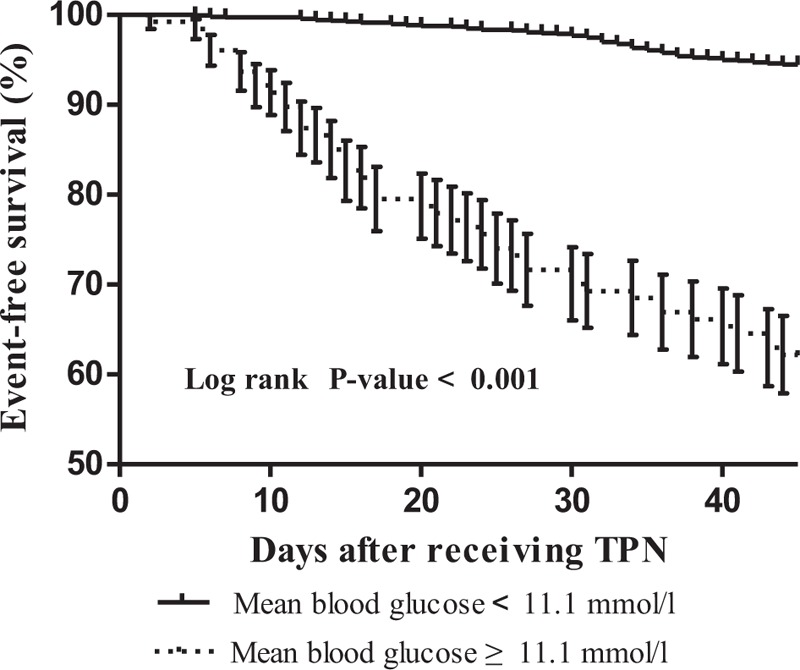
Kaplan–Meier cardiac complication event-free survival curves according to the mean blood glucose level.

The Kaplan–Meier event-free survival curves demonstrated that patients with mean blood glucose level ≥11.1 mmol/L had worse cardiac complications event-free survival than those with mean blood glucose level <11.1 mmol/L during 45 days after receiving TPN (χ^2^ = 203.1, *P* < .001) (Fig. 2). The Kaplan–Meier curves showed that patients with mean blood glucose level ≥11.1 mmol/L had a significantly increased risk of cardiac complications.

## 4 Discussion

The present study demonstrated that the mean glucose levels were significantly higher in elderly medical and surgical nondiabetic patients with cardiac complications than in those without cardiac complications during the 45-day follow-up period. Moreover, the logistic regression analysis showed that higher mean glucose level was an independent predictor of cardiac complications after adjusting for age, sex, comorbidities, and medications. The Kaplan–Meier curves showed that patients with mean blood glucose level ≥11.1 mmol/L had a significantly increased risk of cardiac complications. Therefore, the assessment of the mean blood glucose level during the days after receiving TPN would be useful in predicting cardiac complications.

TPN can ensure the early and consistent delivery of nutrition when enteral nutrition is contraindicated. Hyperglycemia is associated with adverse outcomes in patients receiving TPN.^[2–5,7,9–11]^ Furthermore, the degree of hyperglycemia occurring during TPN treatment is associated with increased adverse outcomes, including cardiac complication in patients receiving TPN.^[11]^ In agreement with these reports, we found a strong correlation between TPN-induced hyperglycemia and poor cardiac clinical outcome in critically and noncritically ill elderly patients without a history of diabetes. The result of the present study strengthened the possible role of TPN-associated hyperglycemia on cardiac adverse outcomes.

The risk factors for developing hyperglycemia responses to TPN in the absence of diabetes included age, rate of dextrose administration, infection, severity of illness, and the interaction of these factors.^[7,9,13,14]^ Age-related changes include minor increases in fasting blood glucose levels and impaired tolerance of glucose loads, and these changes interact with those that follow injury.^[15]^ Moreover, hyperglycemia was associated with high-rate, continuous infusion of TPN dextrose, and excess caloric administration.^[16,17]^ Glucose load has been considered the main factor in developing hyperglycemia during PN, and other factors are also known to predispose to this complication.^[18]^ PN formulations that exceeded a dextrose administration rate of 4 mg/kg/min resulted in more hyperglycemic events.^[19]^ The guidelines of the American Society of Parenteral and Enteral Nutrition (ASPEN) for stressed patients recommend a TPN dextrose infusion rate of no more than 5 to 7 mg/kg/min to prevent an increased risk of hyperglycemia.^[20]^ Additionally, the acute metabolic stress response itself is a major contributor to hyperglycemia in patients without a prior diagnosis of diabetes.^[21]^ Insulin resistance is common in stressed hospitalized patients whose insulin production may be inadequate to compensate for high dextrose infusion rates.^[22]^ The homeostatic mechanisms controlling serum glucose may be lacking in frail elderly patients, and further impairing their ability to react to stress-induced hyperglycemia.^[23]^ Furthermore, counter-regulatory hormones such as catecholamines, glucagon, and corticosteroids are enhanced during PN, probably resulting in glucose metabolism disturbances.^[24]^ In this retrospective study, determining to what extent each of these mechanisms contributed to hyperglycemia was not possible.

Several possibilities might explain the increased risk of cardiac complications with TPN-associated hyperglycemia. Hyperglycemia has been shown to have deleterious effects on the vascular, hemodynamic systems, and prothrombotic shift.^[25,26]^ Coronary collateral blood flow has been reduced with hyperglycemia and then infarct size was increased.^[27]^ Additionally, hyperglycemia promoted apoptosis in some cell systems and that this might contribute to cardiac myocyte loss.^[28]^ Hyperglycemia also increases counter-regulatory hormones, inflammatory cytokines, and oxidative stress,^[29,30]^ which can lead to endothelial dysfunction and cardiovascular complications.^[31]^ In particular, it may also impair fibrinolysis and platelet function,^[32]^ as well as enhanced platelet activation,^[33]^ which may be prothrombotic, lead to hypercoagulability and increase risk for thrombotic events. The combination of these factors is likely to contribute to the increased risk of cardiac complications in a particularly vulnerable population. Consequently, a strong link between TPN-associated hyperglycemia and adverse cardiac outcomes might exist, which suggested that tight glucose control might be important. Therefore, low-calorie PN, perhaps in conjunction with careful and appropriately monitored insulin administration, may reduce or prevent the occurrence of hyperglycemia and be effective in reducing hyperglycemia-associated cardiac complications. Provision of protein and fat via TPN or EN may contribute to reducing the incidence of hyperglycemia, which precludes further investigation. Further prospective, randomized controlled research should focus on the risks and benefits of glycemic control via decreased glucose infusion rates and/or insulin infusion, and assess the relationship between hyperglycemia and cardiac adverse outcomes.

The novel aspect of this study is the population analyzed, which consists of elderly patients with multiple comorbidities, baseline functional status impairment, and a heterogeneous diagnosis. Moreover, as we could not separate the critically ill from the noncritically ill, the cardiac complications in the present study may have been a result of preexisting conditions and the elderly population. Careful surveillance for hyperglycemia after initiating TPN is essential in elderly patients even when previous blood glucose values were within the normal range. If clinically appropriate, a transition should be made from parenteral to enteral nutrition. On the other hand, the definition of hyperglycemia varies among different studies. On the basis of our experience and according to other study,^[34]^ the cut-off value of glucose level 11.1 mmol/L was used. Further research is needed to pinpoint the optimal glucose level for identifying those at higher risk of adverse cardiac outcomes.

Several limitations were noted in the present study. First, the main limitation of this study was its retrospective character and inclusion of heterogeneous groups. The differences in the risk factors other than those included in the multivariate model, such as severity of disease and aggravation of the medical condition, may have affected the results. Second, the type of TPN formula used was not considered, and the ratio and dose of calories administered as dextrose may be not standardized. Third, the study did not address the question of whether treatment of hyperglycemia could reduce the cardiac complications associated with hyperglycemia in patients without a history of diabetes. Moreover, the role of insulin could not be assessed. Further prospective and randomized clinical trials are needed to determine whether the control of blood glucose can improve outcomes in this population. Fourth, patients were collected from 2 hospitals with different protocols for monitoring and controlling blood glucose. These factors were not evaluated in the analysis. Fifth, because the diagnosis of diabetes in our study was based on clinical history, we had no information on the systematic screening for diabetes or impaired glucose tolerance. It was possible that some of the nondiabetic subjects had undiagnosed diabetes. In addition, our study did not detect a difference among the groups in clinical outcomes, such as infection and length of stay. Our observation also had a short duration. Lastly, our cohort was population based and drawn from 2 different hospitals in North China. Therefore, our findings may not be completely representative of the population of other locations with different TPN and glucose control practices. Nevertheless, we consider that these limitations influencing the main findings to be improbable.

In conclusion, our study showed that TPN-induced hyperglycemia was associated with increased risk of cardiac complications in both critically and noncritically ill elderly patients without a history of diabetes. This results suggest that mean blood glucose levels may serve as a simple marker to stratify the risk for optimal triage and management in these patients.
